# Erythroderma as first manifestation of squamous cell lung cancer: rare case report^☆^^☆☆^

**DOI:** 10.1016/j.abd.2019.05.004

**Published:** 2019-12-31

**Authors:** Jorge Arandes-Marcocci, Maribel Iglesias-Sancho, Núria Setó-Torrent, María Teresa Fernández-Figueras

**Affiliations:** Hospital Universitari Sagrat Cor-Grupo Quirónsalud, Barcelona, Spain

**Keywords:** Carcinoma, squamous cell, Dermatitis, exfoliative, Lung neoplasms, Paraneoplastic syndromes, Skin diseases

## Abstract

Erythroderma as the first manifestation of a solid organ malignancy is rare. The underlying cancer is a challenging condition to diagnose. There are a few cases of erythroderma in cancer patients reported in the literature. We here describe the case of a 70-year-old man who presented with asthenia, weight loss, dry cough and total body erythema with desquamation over the past month. A chest computed tomography scan showed a nodular lesion, which was finally diagnosed as a squamous cell lung carcinoma. To our knowledge, as an erythroderma presentation, only 13 cases have been reported in the literature. This case report demonstrates the need to search for a neoplasm in patients presenting with erythroderma, particularly in the presence of accompanying debilitating symptoms.

## Introduction

Erythroderma is characterized by diffuse erythema and scaling of the skin involving 90% or more of the total body surface area. It appears as a cutaneous manifestation of a variety of diseases including solid tumors.^1^ Appropriate criteria (Curth's postulates) are needed to establish a relationship between an underlying malignancy and a cutaneous disorder, but not all individual criterion must be fulfilled to define an association between the skin disease and the neoplasm.^2^ In relation to lung cancer, the most frequently associated paraneoplastic syndromes are hypertrophic osteoarthropathy, besides endocrine and neurological manifestations.^3^ Although lung neoplasms cutaneous expression is relatively atypical, tripe palms, erythema gyratum repens, hypertrichosis lanuginosa acquisita, and Bazex syndrome are the most common paraneoplastic dermatoses.^4^ To our knowledge, erythroderma as the first manifestation of lung cancer is very rare and only 13 cases have been previously reported. Awareness of the relationship between erythroderma and lung neoplasms is crucial for decreasing diagnostic delays and improving oncological outcomes.

## Case report

**Figure 1:**
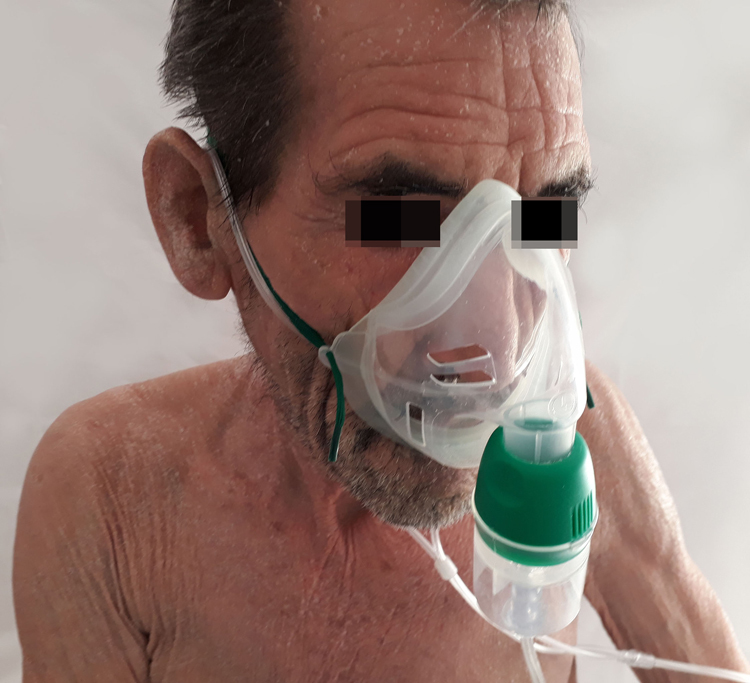
Erythematous rash with fine desquamation onto the face on initial presentation.

**Figure 2:**
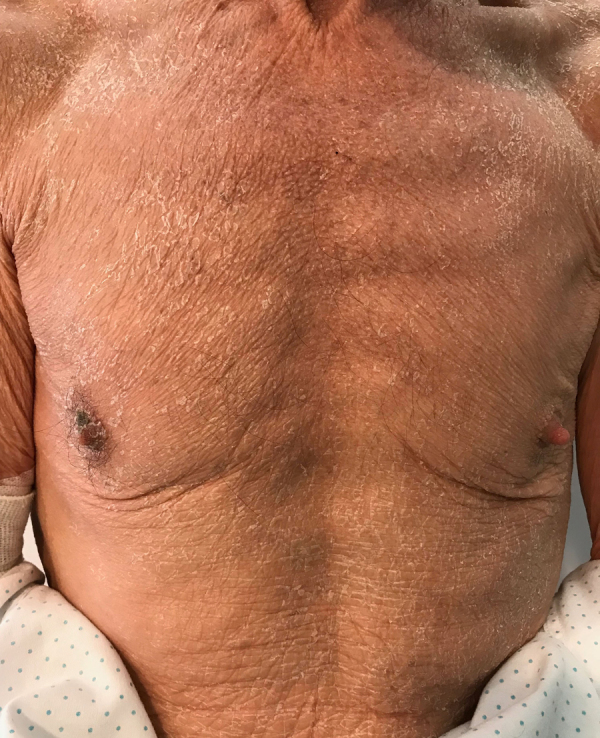
Erythematous rash with fine desquamation onto the trunk on initial presentation.

**Figure 3:**
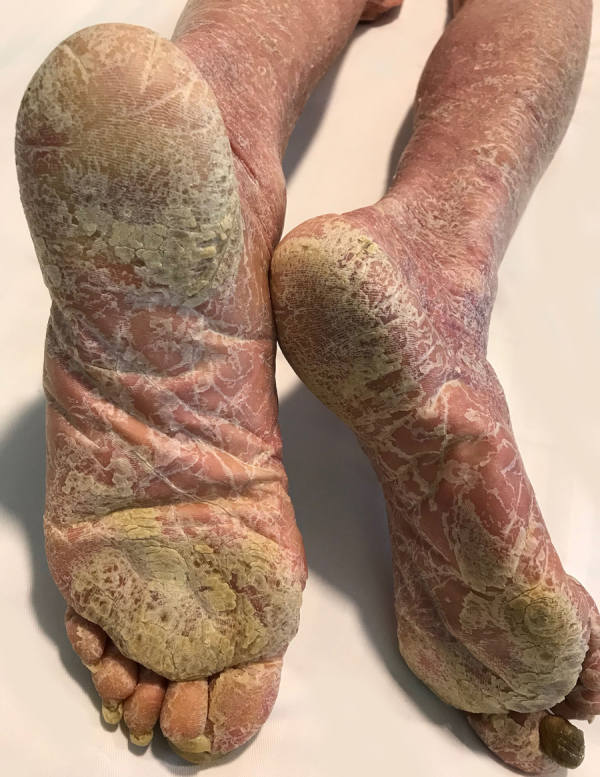
Bilateral leg erythema and plantar keratoderma.

A 70-year-old man, current smoker of 2/3 packs a day, presented to the emergency department with a one-month history of asthenia, weight loss, episodic fever (39 °C), dry cough, instability and a generalized pruritic erythema. He did not refer previous dermatoses and any recent medication intake. Physical examination revealed a total body erythema with fine scales (Figure 1, Figure 2), a plantar keratoderma (Fig. 3), and a left axillary lymphadenopathy. The rest of the clinical examination was unremarkable. Routine blood test, including a full blood count, electrolytes and liver function only revealed leucocytosis (white cell counts 15,250 μL; normal range 3500–1000) at the expense of neutrophils (84.7%) and elevated C-reactive protein of 9.9 mg/dL (normal <1 mg/dL). The X-ray film was unrevealing.

**Figure 4:**
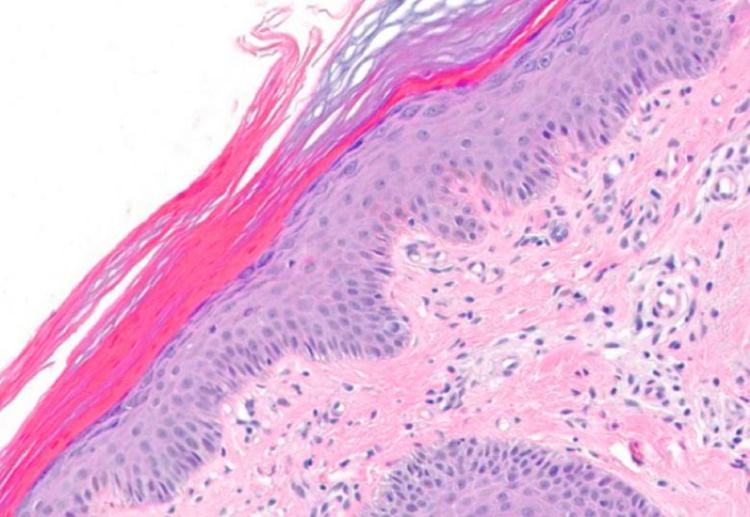
The biopsy showed a mild spongiotic and psoriasiform changes with discrete follicular hyperkeratosis (Hematoxylin & eosin x20).

**Figure 5:**
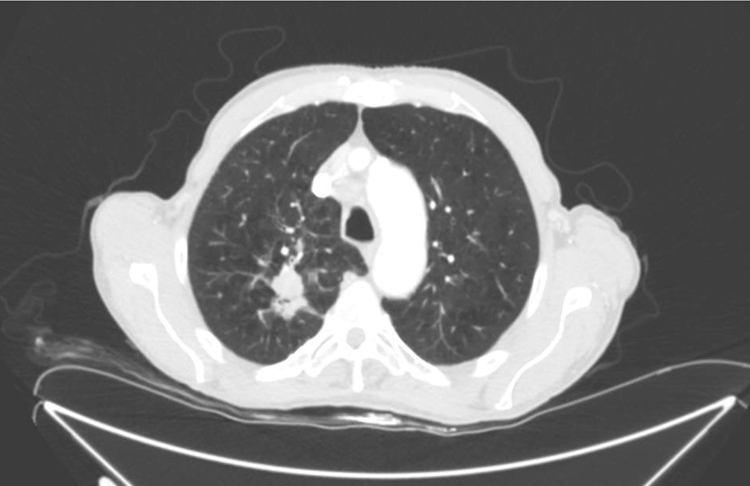
Computed tomography scan findings: 22 × 34 mm nodular lesion with spiculated borders on the upper right lobe with right supraclavicular, hilar, precaval and subcarinal lymphadenopathies.

Two punch biopsies and a new blood test were taken, including serum protein levels, viral serologies, blood smear, muscle enzymes, Sézary cells, flow cytometry, and immunological and tumoral markers. The remainder of the laboratory findings were within normal limits (including serum aldolase) except of Creatin phosphokinase (CPK) (1.309 IU/L; normal range 1–175 IU/L) and creatine kinase-MB (CK-MB) (27.3 IU/L; normal range 0–20 IU/L). Histological specimens revealed mild spongiotic and psoriasiform changes with discrete follicular hyperkeratosis, as well as nonspecific histopathology features (Fig. 4). We started a supportive treatment with emphasis on hyperproteic diet; temperature and hydration control in addition to topical steroids. Because of the new-onset of weight loss and concern for underlying malignancy as a potential cause for his erythroderma, a thorough workup for occult malignancy was completed. A chest computed tomography revealed 22 × 34 mm nodular lesion on the upper right lobe with multiple lymphadenopathies (Fig. 5), being the histopathology compatible with a squamous cell carcinoma. Head computed tomography demonstrated two occipital metastatic lesions, being finally classified as IV stage (T2N3M1). At 6 weeks, the patient's rash was similar to that at the initial presentation, however less itchy. The patient died after 3 months of the diagnosis due to metastatic cancer progression.

## Discussion

Pre-existing dermatoses, particularly psoriasis and eczema, as well as drug reaction and T-cell lymphomas have been identified as common aetiologies of erythroderma. In some patients, however, the cause is unknown. Patients with idiopathic erythroderma should be closely followed over prolonged time periods, with multiple biopsies since a significant percentage of these cases will progress to cutaneous T-cell lymphoma.^5^, ^6^

The histopathology often reveals non-specific changes. Even so, the majority of the studies consider necessary the biopsy, because in 43–66% of the cases it was helpful to establish a definite diagnosis.^6^ Erythroderma is a dermatologic emergency; temperature, hydration, decreased serum proteins and heart failure needs to be corrected and monitored. It is important to consider the underlying etiology to establish the target treatment and any potential drugs must be stopped.^7^

Paraneoplastic erythroderma is most commonly associated with lymphoproliferative disorders, other than mycosis fungoides and Sézary syndrome. However, it can also be an expression of a solid cancer, usually in a late stage of the disease,^8^ as in the case here reported. Paraneoplastic syndromes can appear before, during or after the tumor diagnosis. The physiopathology is not well understood. However, the large majority of the skin findings are inflammatory or proliferative, and can also occur in the absence of the malignancy condition.

As shown in other cases published in the literature, normally the paraneoplastic expression modifies itself with the tumor regression or progression. Only 1% of internal malignancies can provide the first clues for a diagnosis through skin expression.^9^ In our case, we could not see changes on the erythroderma given that lung cancer could not be treated because of the patient's death. Therefore, following Curth's postulates, we can relate but not definitely classify the erythroderma as paraneoplastic syndrome.

Among all of the paraneoplastic dermatoses reported in the literature, the erythroderma is one of the less mentioned. Additionally, it is non-specific of an internal malignancy, making it difficult to guide toward a clear etiology. Erythroderma can also present as a cutaneous finding associated with dermatomyositis.^10^ In our case, the lack of typical skin findings (heliotrope rash on the eyelids and Gottron's sign and/or papules) and the lowering of the CPK and CK-MB levels in the follow-up, made us to exclude the diagnosis of dermatomyositis.

In conclusion, in those patients with concomitant history of insidious development, progressive decompensation and refractory to standard therapies, clinicians should perform further investigations. Hence, it may result in early detection of potentially treatable malignancy.

## Financial support

None declared.

## Authors’ contribution

Jorge Arandes Marcocci: Approval of the final version of the manuscript; conception and planning of the study; elaboration and writing of the manuscript; collection, analysis, and interpretation of data; effective participation in research orientation; intellectual participation in the propaedeutic and/or therapeutic conduct of the studied cased; critical review of the literature; critical review of the manuscript.

Maribel Iglesias-Sancho: Approval of the final version of the manuscript; conception and planning of the study; elaboration and writing of the manuscript; collection, analysis, and interpretation of data; critical review of the literature; critical review of the manuscript.

Núria Setó-Torrent and María Teresa Fernández-Figueras: Conception and planning of the study; effective participation in research orientation; critical review of the literature; critical review of the manuscript.

## Conflicts of interest

None declared.
